# Postnatal Cadmium Exposure, Neurodevelopment, and Blood Pressure in Children at 2, 5, and 7 Years of Age

**DOI:** 10.1289/ehp.0900765

**Published:** 2009-05-26

**Authors:** Yang Cao, Aimin Chen, Jerilynn Radcliffe, Kim N. Dietrich, Robert L. Jones, Kathleen Caldwell, Walter J. Rogan

**Affiliations:** 1 Epidemiology Branch, National Institute of Environmental Health Sciences, National Institutes of Health, Department of Health and Human Services, Research Triangle Park, North Carolina, USA; 2 Department of Health Statistics, Faculty of Health Services, Second Military Medical University, Shanghai, China; 3 Department of Preventive Medicine and Public Health, Creighton University School of Medicine, Omaha, Nebraska, USA; 4 Children’s Hospital of Philadelphia and University of Pennsylvania School of Medicine, Philadelphia, Pennsylvania, USA; 5 Department of Environmental Health, Division of Epidemiology and Biostatistics, University of Cincinnati College of Medicine, Cincinnati, Ohio, USA; 6 Inorganic and Radiation Analytical Toxicology Branch, Centers for Disease Control and Prevention, Atlanta, Georgia, USA

**Keywords:** behavior, blood pressure, cadmium, children, clinical trial, intelligence, neurodevelopment

## Abstract

**Background:**

Adverse health effects of cadmium in adults are well documented, but little is known about the neuropsychological effects of cadmium in children, and no studies of cadmium and blood pressure in children have been conducted.

**Objective:**

We examined the potential effects of low-level cadmium exposure on intelligence quotient, neuropsychological functions, behavior, and blood pressure among children, using blood cadmium as a measure of exposure.

**Methods:**

We used the data from a multicenter randomized clinical trial of lead-exposed children and analyzed blood cadmium concentrations using the whole blood samples collected when children were 2 years of age. We compared neuropsychological and behavioral scores at 2, 5, and 7 years of age by cadmium level and analyzed the relationship between blood cadmium levels at 2 years of age and systolic and diastolic blood pressure at 2, 5, and 7 years of age.

**Results:**

The average cadmium concentration of these children was 0.21 μg/L, lower than for adults in the National Health and Nutrition Examination Survey (NHANES), but comparable to concentrations in children < 3 years of age in NHANES. Except for the California Verbal Learning Test for Children, there were no differences in test scores among children in different cadmium categories. For children with detectable pretreatment blood cadmium, after adjusting for a variety of covariates, general linear model analyses showed that at none of the three age points was the coefficient of cadmium on Mental Development Index or IQ statistically significant. Spline regression analysis suggested that behavioral problem scores at 5 and 7 years of age tended to increase with increasing blood cadmium, but the trend was not significant. We found no significant associations between blood cadmium levels and blood pressure.

**Conclusion:**

We found no significant associations between background blood cadmium levels at 2 years of age and neurodevelopmental end points and blood pressure at 2, 5, and 7 years of age. The neuropsychological or hypertensive effects from longer background exposures to cadmium need further study.

Exposure to toxic metals is a health hazard throughout the world today. In addition to lead and mercury, cadmium has been identified as one of the most probable causes of disease related to heavy metal exposure observed in primary care medicine (Hu 2000). Both animal experiments and epidemiologic studies have confirmed that cadmium is toxic to kidney, liver, bone, and gonads and causes cancer (Alfven et al. 2002; Buchet et al. 1990; Mandel et al. 1995; Parizek and Zahor 1956; Rikans and Yamano 2000). The Agency for Toxic Substances and Disease Registry (ATSDR) has listed cadmium among the top seven of the 275 most hazardous substances in the environment (ATSDR 1999). The placenta acts as a barrier for cadmium, in contrast to lead and mercury, and very little cadmium (< 10%) is transferred to the fetus (Osman et al. 2000). Also, the transfer of cadmium to milk is low (Hallen et al. 1995). Thus, the fetus and the newborn are protected against cadmium. Cadmium exposure, however, can start at a very young age. The main exposure sources for children are food, environmental tobacco smoke, and house dust. Cadmium can enter the blood by absorption from the stomach or intestines after ingestion of food or water or by absorption from the lungs after inhalation. Approximately 50% of total body stores are in the liver and kidney (Pope and Rall 1995). Once cadmium enters the body, it has a biological half-life of 10–30 years in kidney and 5–10 years in liver [U.S. Environmental Protection Agency (EPA) 1997; International Programme on Chemical Safety 1992]. The main effects of chronic cadmium toxicity, renal disease and bone loss, are related to cadmium concentration in the kidney and the consequent alteration of renal function.

Animal experiments have reported neurotoxic and behavioral effects of cadmium (Gupta et al. 1991). The neurotoxicity and developmental toxicity of cadmium were also observed in human children in several epidemiologic studies conducted in the 1970s and 1980s. In all of these studies, the biomarker of exposure was the concentration of cadmium in hair. In case–control studies, higher concentrations of cadmium were reported in children with mental retardation (Jiang et al. 1990; Marlowe et al. 1983) and with learning difficulties or dyslexia (Capel et al. 1981; Pihl and Parkes 1977). A significant inverse relationship was described between *in utero* exposure to cadmium and lead and the child’s motor and perceptual abilities at 6 years of age (Bonithon-Kopp et al. 1986). In a cohort study, the concentration of cadmium was inversely related to IQ (Thatcher et al. 1982). Other investigators reported associations between cadmium and children’s performance on verbal IQ and visual-motor and cognitive tasks (ATSDR 1999; Marlowe et al. 1985b; Moon et al. 1985). However, these studies have received little attention, and it is not clear whether the effects described were due to exposure to cadmium or to other substances (i.e., lead exposure).

Effects of cadmium exposure on the cardiovascular system and cadmium-induced hypertension have also been reported in human studies and animal models (Satarug et al. 2005, 2006; Staessen et al. 1992), but epidemiologic studies are inconsistent. Although some studies found positive associations (McKenzie and Kay 1973; Pizent et al. 2001; Tellez-Plaza et al. 2008; Vivoli et al. 1989; Whittemore et al. 1991), other studies found null or even inverse associations (Beevers et al. 1976; Kagamimori et al. 1986; Staessen et al. 1984, 1991; Telisman et al. 2001). Most of these studies were limited by small sample sizes, lack of adjustment for potential confounders, no standardization of blood pressure measurements, and other methodological constraints (Tellez-Plaza et al. 2008). No epidemiologic studies of cadmium and blood pressure in children have been conducted to date.

The Treatment of Lead-Exposed Children (TLC) trial (Dietrich et al. 2004) provided a unique opportunity to examine several important questions for the first time: the associations of cadmium with IQ, neuropsychological function, and behavior in children, using blood cadmium as a measure of exposure, and the association of cadmium with blood pressure in children from a large-scale study.

## Subjects and Methods

### Study design

The TLC study was a multi-center, placebo-controlled, randomized clinical trial that examined the effect of succimer treatment on growth, behavior, and development of lead-exposed children in the United States. The study was conducted between September 1994 and June 2003 in Philadelphia, Pennsylvania; Newark, New Jersey; Cincinnati, Ohio; and Baltimore, Maryland. The study was approved by the institutional review boards (IRBs) at the clinical sites, data coordinating center (Harvard University, Cambridge, MA, USA), and the National Institute of Environmental Health Sciences. TLC complied with all applicable U.S. regulations; written informed consent was obtained from all children’s parents or guardians. For the present study, we created an anonymized data set with no links to individual identifiers but with clinical and developmental information linked to the samples. Analysis of those samples for cadmium and analysis of data using the results were declared exempt from further IRB clearance by the Office of Protection from Research Risks at the National Institutes of Health.

Children were eligible for the initial TLC visit if they were expected to be between 12 and 33 months of age at randomization and had a referral blood lead level between 20 and 44 μg/dL. A total of 780 children entered the randomization phase, with 396 children allocated to succimer (Chemet; McNeil Laboratories, Fort Washington, PA, USA) and 384 allocated to placebo. Depending on the blood lead concentration, children in the succimer group could receive up to three 26-day courses of treatment; the number of courses given to placebo children was frequency-matched to that in the succimer group. Blood samples were collected on days 7, 28, and 42 after the beginning of each of three treatment courses, as well as at every 3 months through the 24-month posttreatment follow-up, and then every 4 months until 72 months of age or the end of the study. Details of the study design and participant flow of the TLC study have been published elsewhere (Dietrich et al. 2004; TLC Trial Group 1998).

### Neuropsychological and behavioral tests

Before treatment began, we administered the Bayley Scales of Infant Development-II (BSID-II) (Bayley 1993), the most widely used measure of infant intelligence. The Mental Development Index (MDI) of the BSID-II is analogous to an IQ score. After treatment, two waves of cognitive, behavioral, psychological, and developmental assessments were administered at about 5 and 7 years of age. The child’s IQ was assessed with the Wechsler Preschool and Primary Scales of Intelligence–Revised (Wechsler 1989) and Wechsler Intelligence Scale for Children–III (Wechsler 1991) at 5 and 7 years of age, respectively. The neuropsychological and behavioral tests used at 5 years of age included the Developmental Neuropsychological Assessment (NEPSY) (Korkman 1998) and Conners’ Parent Rating Scale–Revised(CPRS-R) (Conners 1997). At 7 years of age, the NEPSY, Conners’ Continuous Performance Test (CPT) (Conners 1995), California Verbal Learning Test for Children (CVLTC) (Delis et al. 1994), Woodcock Language Proficiency Battery–Revised (WLPB-R) (Woodcock 1991), Neurologic Examination for Subtle Signs (NESS) (Denckla 1985), Behavioral Assessment System for Children teacher rating scale (BASC-TRS), and BASC parent rating scale (BASC-PRS) (Reynolds and Kamphaus 1992) were administered. The detailed information of the use of these standardized instruments in the TLC have been described elsewhere (Dietrich et al. 2004; Rogan et al. 2001).

### Measurement of blood cadmium concentration

The Division of Laboratory Sciences at the National Center for Environmental Health at Centers for Disease Control and Prevention (CDC) analyzed the total blood cadmium levels using samples drawn about 1 week before randomization (pretreatment) and samples drawn 1 week after treatment began (posttreatment), which showed the largest mean difference between treatment groups in blood lead level. Whole blood specimens were tested using inductively coupled plasma mass spectrometry (ICP-MS) (CDC 2007). This multielement analytical technique is based on quadrupole ICP-MS technology (Date and Gray 1989). The limit of detection (LOD) of cadmium was 0.2 μg/L.

For cadmium analysis, National Institute of Standards and Technology (NIST) standard reference materials and two levels of in-house blood pools traceable to the reference material were used for daily quality control. One of two different levels of a blind quality control material was inserted in every analytical group of samples for an additional quality control check. NIST Standard Reference Material 966 (NIST, Gaithersburg, MD, USA) was analyzed periodically to ensure analytical accuracy.

### Blood pressure measurements

At each visit during the treatment courses and follow-up, study nurses measured systolic and diastolic blood pressures for children. A Dinamap Vital Signs Monitor (an automatic device; Critikon, Inc., Tampa, FL, USA) was used for all blood pressure measurements. Blood pressure was measured when children were seated, and the average of up to three measurements per visit was used for statistical analysis (Chen et al. 2006).

### Statistical analysis

Because > 40% of the pre- and posttreatment blood cadmium levels were below the LOD, we treated them as heavily left-censored data to conduct summary analyses. Instead of simply replacing the nondetectable observations with LOD divided by the square root of 2, we read the nondetectable values as being between zero and LOD (the “censoring interval”) and then used a maximum likelihood estimation method to estimate mean, percentiles, and variance. Censored data analyses were conducted with Minitab software (version 15.1; Minitab, Inc., State College, PA, USA). For geometric mean (GM) estimation, however, we used the method of replacing nondetectable values with LOD divided by the square root of 2. Samples not measured because of insufficient sample volume were excluded from all analyses.

The correlations between blood cadmium, blood lead, IQ, and behavioral indices were tested by nonparametric Kendall’s tau-b correlation coefficient (ktau-b), which makes no assumption of linearity between variables and accommodates data sets with censored values (Helsel 2004).

To examine the associations between cadmium and neuropsychological functions, we grouped children into five categories based on the LOD of cadmium and the four quartiles of detectable pretreatment cadmium. The differences of neuropsychological and behavioral test scores at 5 and 7 years of age across cadmium categories were compared by Dunnett’s two-tailed *t*-test, with the blood cadmium < LOD category as reference group.

Because the large percentage of censored data will distort the compliance for the distribution requirement, and because we would not expect an association between IQ and lower cadmium levels if there was no association between IQ and higher cadmium levels, we further limited the analysis to the children with detectable blood cadmium and analyzed the relationships between cadmium exposure and IQ and behavioral test scores. In these children, the blood cadmium concentrations were lognormally distributed, so we used a logarithmic transformation of the exposure variable. We also examined the cadmium and IQ and behavior associations using scatter plots and cubic smoothing splines (which provided a fitted curve constructed by piecewise polynomials) with S-PLUS software (version 8.0; Insightful Corp, Seattle, WA, USA). Because spline regressions showed an approximately linear relation, we used a general linear model (GLM) to examine the cadmium effect. Based on the literature and our previous work with these data (Bonithon-Kopp et al. 1986; Chen et al. 2007; Marlowe et al. 1985a), *a priori* covariates included exact age at IQ measurement, treatment group, caregiver’s IQ, clinical center, single parent (yes or no), language (English or Spanish), race (non-Hispanic black or other), sex, parents’ employment (neither working or either working), parents’ education (< 12 years, 12 years, or > 12 years), and concurrent blood lead levels. The same set of covariates was included for the regression models at 2 years of age (except for treatment group) and at 5 and 7 years of age. Treatment per se was not associated with cadmium, IQ, or behavior scores in GLM analyses (Dietrich et al. 2004; Rogan et al. 2001), and additional adjustment for treatment group did not markedly change the results; thus, the groups were combined in subsequent analyses.

The relationship between blood cadmium and blood pressure was examined by local polynomial regression—a nonparametric regression method that makes no assumptions about the functional form for the expected value of a response given a regressor (Fan and Gijbels 1996).

All models were examined for statistical outliers and influential points. Each model was run first with outliers and then without outliers, and the results were compared. All of the results were essentially the same with or without outliers. Correlation and GLM analyses were done by STATA software (version 10.1; StataCorp., College Station, TX, USA) and SAS software (version 9.13; SAS Institute Inc., Inc., Cary, NC, USA).

## Results

Of 780 children randomized in the parent study, 767 had sufficient pretreatment sample available for cadmium analysis. Six hundred eighty-three children had posttreatment samples, and 675 had sufficient sample available for cadmium analysis. Blood cadmium was detected and quantified in 441 (57%) pretreatment samples and in 373 (55%) posttreatment samples. Pretreatment blood cadmium and lead levels were not correlated (ktau-b = 0.04, *p* = 0.11), which suggests that cadmium exposure was independent of lead exposure.

**Figure 1:**
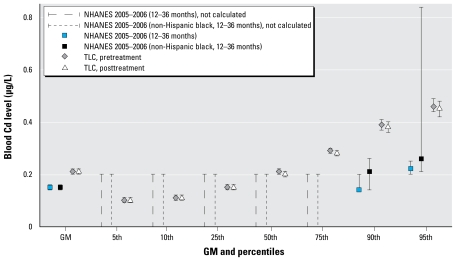
Comparison of blood cadmium levels (95% CIs) between TLC and NHANES children, based on the data from NHANES 2005–2006 (CDC 2009). The cadmium values for the 5th to 75th percentiles from NHANES were not calculated because proportion of results below the LOD was too high to provide a valid result.

For all children, the GMs and 95% confidence intervals (CIs) of pre- and posttreatment cadmium concentrations of placebo group were 0.21 μg/L (0.20–0.22 μg/L) and 0.20 μg/L (0.19–0.21 μg/L), respectively; the GMs and 95% CIs of pre- and posttreatment cadmium concentrations of succimer group were 0.21 μg/L (0.20–0.22 μg/L) and 0.21 μg/L (0.20–0.22 μg/L), respectively. The censored regression analysis showed that there was no significant difference of cadmium concentrations between placebo group and succimer group (*p* = 0.79 and *p* = 0.58 for pre- and posttreatment, respectively). These levels were lower than observed in National Health and Nutrition Examination Survey (NHANES) adults (GM = 0.41 μg/L; 95% CI, 0.38–0.45 μg/L) (CDC 2005) but comparable to NHANES children < 3 years of age (Figure 1).

Table 1 shows scores for neuropsychological and behavioral tests by cadmium levels at 5 and 7 years of age. Not all children completed all tests, so the sample sizes vary slightly. For each outcome domain of a specific test instrument, cadmium categories with measureable levels were compared with the reference (< LOD) category. Except for the list A memory and learning slope (the positive learning slope over five verbal memory recall trials, with the steeper positive value the more favorable indicator of learning) of CVLTC, these test scores did not differ among cadmium categories.

**Figure 2:**
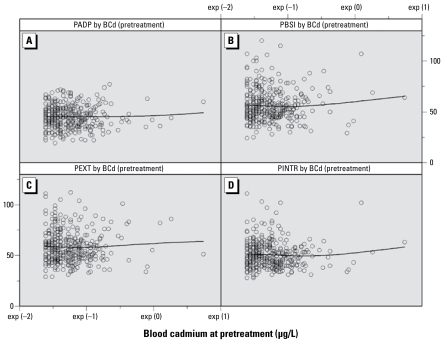
Scatterplots and smoothed spline regression curves of pretreatment blood cadmium (BCd) concentrations and parent-rated BASC test scores at 7 years of age: PADP (*A*), PBSI (*B*), PEXT (*C*), and PINTR (*D*) denote parent-rated BASC scores of adaptive skills, behavioral symptoms index, externalizing problems, and internalizing problems, respectively.

After excluding the nondetectables, the neuropsychological and behavioral test scores still did not show significant associations with blood cadmium concentration (data not shown). Although not statistically significant, the four-knot cubic spline regression analysis showed that in the models with behavioral problem scores, both the first-order coefficient and the third-order coefficient of blood cadmium were positive, and the absolute value of the third-order coefficient was larger than the absolute value of the second-order coefficient (data not shown), which suggests that behavioral problem scores at 5 and 7 years of age tend to increase (which indicates worse behavior) with increasing blood cadmium. When three extreme cadmium values were omitted, the trend persisted in results at 7 years of age (Figure 2).

Table 2 shows the baseline and follow-up demographic characteristics for children with detectable pretreatment blood cadmium. Except for 1-μg/dL differences in blood lead levels at 5 and 7 years of age, the two groups did not differ and so were combined. We plotted blood cadmium and MDI at 2 years of age and IQ at 5 and 7 years of age and did spline regression. MDI showed a weak increasing trend by cadmium levels, but at 5 and 7 years of age IQ showed a weak decreasing trend by cadmium levels (data not shown). Based on the approximately linear relationship, we conducted GLM analyses of cadmium and MDI or IQ at 2, 5, and 7 years of age adjusting for a variety of covariates. The pretreatment cadmium level and posttreatment cadmium level were significantly correlated with each other (ktau-b = 0.45), so we used pretreatment cadmium level as the main independent variable in GLM analyses. In the GLM models, the coefficient of cadmium was positive at 2 years but negative at 5 and 7 years of age, but none of the coefficients was statistically significant (Table 3). GLM analyses were repeated after excluding three extreme cadmium values, and the results were very similar.

**Figure 3:**
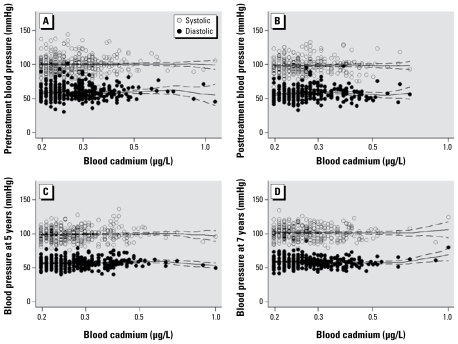
Scatterplots [and local polynomial regression predictions (with 95% CI)] of systolic and diastolic blood pressure for pretreatment (*A*), posttreatment (*B*), 5 years of age (*C*), and 7 years of age (*D*) by detectable blood cadmium concentrations at pretreatment (*A*) and posttreatment (*B*–*D*).

For blood pressure, because no differences between succimer and placebo groups at baseline and other time points were observed (Chen et al. 2006), we combined the two groups to analyze the association between blood cadmium and blood pressure. When treatment began, the mean (± SD) systolic and diastolic blood pressures of children with detectable blood cadmium were 100.3 ± 12.4 mmHg and 59.5 ± 10.1 mmHg, respectively. There was a significant (*p* = 0.03) 1.7-mmHg decrement of the mean systolic blood pressure at 1 week after treatment began, which might be attributable to the significantly decreased blood lead level, but the mean diastolic blood pressure did not change after treatment. We then plotted the cross-sectional association of blood cadmium level and blood pressure of children with detectable blood cadmium with data from pretreatment, posttreatment, and 5 and 7 years of age, along with local polynomial regression predictions (Figure 3). We found no clear association between pre- or posttreatment blood cadmium level and blood pressure at these ages.

## Discussion

Using data from a large clinical trial of children with moderately high lead exposure, we examined the association between cadmium exposure and neurodevelopment in children. TLC children had blood cadmium levels comparable to those of children of the same age in a representative sample of the general U.S. population but significantly lower than levels of the adults in the same national sample. Because cigarette smoke is a major source of cadmium exposure, and these TLC children are exposed only to passive smoking, this lower concentration was expected. We did not find a significant relationship between blood cadmium level and IQ or neuropsychological and behavioral test scores after adjusting for lead levels and other potentially confounding variables. Although the estimates of behavioral test scores of CPRS-R at 5 years of age and of BASC behavioral problems (both teacher and parent rated) at 7 years of age were not significantly associated with detectable blood cadmium level, we did find that behavioral problems tended to increase with increasing blood cadmium concentrations at 7 years of age. At the most, this suggests that cadmium exposure at 2 years of age may have some small neurodevelopmental effect later when a wider range of behavioral issues can be examined, especially with higher or longer exposure.

The commercial use of cadmium has declined in developed countries in response to environmental concerns. In the United States, the GM daily cadmium intake is about 0.4 μg/kg/day, less than half of the U.S. EPA’s oral reference dose (U.S. EPA 2006). Cadmium, however, has a long biological half-life, so even low doses may bioaccumulate and lead to significant adverse effects if exposure continues for a long period of time. Animal experiments showed that cadmium’s most direct effect on brain metabolism is to inhibit a number of sulfhydryl-containing enzymes (Stowe et al. 1972). Consequently, chronic exposure to cadmium has a depressive effect on levels of norepinephrine, serotonin, and acetylcholine (Singhal et al. 1976; Webb 1975). Animal studies also demonstrated cadmium transport through the immature blood–brain barrier of suckling rat pups (Andersson et al. 1997). Although there are few reports on blood–brain barrier and cadmium in human samples, there has been a single case report of cadmium encephalopathy in a 2-year-old child, supporting the notion that the blood–brain barrier does not prevent entry of cadmium in early childhood (Provias et al. 1994). This evidence suggests that neurobehavioral effects during development may also be a sensitive end point for cadmium toxicity in addition to renal dysfunction (Petersson-Grawe et al. 2004).

In the 1970s and 1980s, there were several studies of cadmium and child neuropsychological performance. Based on hair samples from 31 learning disabled and 22 normal children, Pihl and Parkes (1977) found that concentrations of cadmium, cobalt, manganese, chromium, and lithium could classify subjects as learning disabled or normal with 98% accuracy. Our study did not identify children specifically as “learning disabled,” but we found no association between cadmium and IQ or behavior problems, which are often components of learning disability. Hair measures are subject to contamination; if the behavior of the “learning disabled” children leads to contamination, then the finding may be attributable to reverse causality. Capel et al. (1981) found higher cadmium levels in the hair of 73 dyslexic children than in 44 controls. However, these researchers measured nine elements without any specific hypotheses, found several differences, but interpreted their findings as preliminary and in need of confirmation. Thatcher et al. (1982) reported that hair cadmium in 149 children 5–16 years of age was related to intelligence tests, motor function, and school achievement and found that cadmium had a stronger effect on verbal IQ than did lead. Although these researchers used statistical modeling to control confounding due to race, rural residence, and simultaneous lead exposure, the potential for substantial residual confounding remains with this relatively small data set. Bonithon-Kopp et al. (1986) analyzed hair samples from 26 newborn babies and found a significant negative relationship between the prenatal cadmium exposure and child’s motor and perceptual abilities at 6 years of age. These researchers did not collect information on maternal smoking, which is a source of cadmium exposure and is also associated with child’s IQ (Alati 2008). Marlowe et al. (1983) found an association between elevated cadmium concentrations and mild mental retardation and borderline intelligence in 135 children. Another study conducted by Marlowe at al. (1985a) in 80 randomly selected elementary-age children showed synergistic interaction of lead with cadmium related to increased scores on acting-out, disturbed peer relations, and immaturity on the Walker Problem Behavior Identification Checklist. These studies are cross-sectional, and it is difficult to dismiss the possibility that some aspect of the children’s behavior led to their exposure.

All of the above-mentioned studies are case–control or cross-sectional in design, and confounding from other substances (i.e., lead exposure) cannot be excluded entirely. Moreover, in previous studies on cadmium exposure and child neurodevelopment, none of them measured blood cadmium levels but used the concentration of cadmium in hair. Although hair has been used widely in metals exposure studies as an easily accessible material for estimating an accumulated metals body burden, a major criticism of hair analysis is the wide inter-laboratory variation in the results (ATSDR and CDC 2001; Capel et al. 1981), and it may not reflect the appropriate exposure averaging time for the outcomes evaluated. Because cadmium enters the blood quickly and has an extremely long biological half-life (ATSDR 1999), it is reasonable to assume that blood concentrations reflect both recent and cumulative exposures (CDC 2005; Hassler et al. 1983).

Our study has the strength of large sample size, direct cadmium exposure measurement in blood, long follow-up period, high retention rate of subjects in the follow-up, multiple measurements of behavior and cognition, and good quality control in the measurements. The longitudinal nature of the data also allowed us to take a prospective view on the association between postnatal cadmium exposure and child neurodevelopment.

Study limitations include lack of cadmium measurement at 5 and 7 years of age. According to the results based on NHANES data, blood cadmium concentration increases with age (CDC 2005). Further research is needed to better understand the effects of concurrent and cumulative cadmium exposure on children’s cognitive and behavior.

We also examined the association between blood pressure and low-level cadmium exposure. Cadmium induces hypertension in animal models, and the potential mechanism is hypothesized to be related to kidney dysfunction (Satarug et al. 2006). Epidemiologic studies, however, of environmental cadmium exposure and blood pressure are not consistent, and no epidemiologic study on children has measured both blood cadmium and blood pressure levels. In our study, we find no relation between a background cadmium exposure and blood pressure, but a potential hypertensive effect from longer background exposures needs further study.

## Figures and Tables

**Table 1:** Neuropsychological and behavioral test scores at ages 5 and 7 years of age by prepretreatment cadmium level.^a^

	Cadmium level									
	< LOD^b^		LOD–0.22 μg/L		0.23–0.25 μg/L		0.26–0.32 μg/L		≥ 0.33 μg/L	
Neuropsychological/behavioral test	No.	Mean ± SE	No.	Mean ± SE	No.	Mean ± SE	No.	Mean ± SE	No.	Mean ± SE
Age 5										
NEPSY										
Attention and executive	264	88 ± 1.8	88	85 ± 2.3	97	87 ± 2.3	84	86 ± 2.4	91	86 ± 2.3
Language	253	83 ± 1.9	88	82 ± 2.2	95	84 ± 2.3	80	84 ± 2.3	90	82 ± 2.3
Sensorimotor	267	90 ± 1.7	90	88 ± 2.1	99	89 ± 2.2	83	88 ± 2.2	96	90 ± 2.2
Visuospatial	270	93 ± 1.4	92	93 ± 1.7	102	95 ± 1.7	87	92 ± 1.8	99	92 ± 1.7
Memory	268	86 ± 1.7	90	86 ± 2.0	99	88 ± 2.1	82	87 ± 2.2	97	85 ± 2.1
CPRS-R										
Oppositional index	285	63 ± 1.5	99	63 ± 1.9	104	64 ± 1.9	92	65 ± 1.9	103	65 ± 1.9
Hyperactivity index	285	66 ± 1.4	99	65 ± 1.8	104	66 ± 1.8	92	65 ± 1.9	103	65 ± 1.8
ADHD index	285	62 ± 1.4	99	64 ± 1.7	104	63 ± 1.7	92	60 ± 1.8	103	63 ± 1.7
Behavioral index	285	64 ± 1.3	99	64 ± 1.6	104	64 ± 1.6	92	63 ± 1.6	103	64 ± 1.6

Age 7										
NEPSY										
Attention and executive	226	89 ± 1.8	83	89 ± 2.3	85	92 ± 2.4	71	87 ± 2.5	91	88 ± 2.3
CPT d-Prime	213	55 ± 1.2	81	55 ± 1.4	84	54 ± 1.5	71	53 ± 1.5	89	56 ± 1.4
CVLTC										
List A memory	245	41 ± 1.2	92	41 ± 1.5	94	44 ± 1.6*	79	41 ± 1.6	96	41 ± 1.6
Learning slope	245	−0.5 ± 0.1	92	−0.1 ± 0.2*	94	−0.3 ± 0.2	79	−0.3 ± 0.2	96	−0.4 ± 0.2
WLPB-R										
Broad reading score	228	92 ± 2.1	85	93 ± 2.6	85	93 ± 2.7	72	91 ± 2.8	93	92 ± 2.6
CPT Hit response time	213	42 ± 1.5	81	44 ± 1.9	84	46 ± 1.9	71	40 ± 2.0	89	46 ± 1.9
NESS										
Sequential movement time	213	1.1 ± 0.1	82	1.1 ± 0.2	82	0.9 ± 0.2	68	1.3 ± 0.2	66	1.0 ± 0.2
BASC-TRS										
Adaptive skills	205	48 ± 1.1	71	47 ± 1.4	79	48 ± 1.6	65	48 ± 1.6	80	47 ± 1.5
Behavioral Symptoms Index	206	52 ± 1.4	76	51 ± 1.8	80	52 ± 1.8	68	52 ± 1.9	80	52 ± 1.8
Externalizing problems	205	52 ± 1.6	75	51 ± 2.0	81	51 ± 2.0	67	50 ± 2.1	81	53 ± 2.0
Internalizing problems	206	52 ± 1.2	76	51 ± 1.6	81	52 ± 1.6	68	51 ± 1.7	80	51 ± 1.6
School problems	206	55 ± 1.4	75	55 ± 1.8	81	55 ± 1.9	68	54 ± 2.0	81	55 ± 1.9
BASC-PRS										
Adaptive skills	246	43 ± 1.2	92	42 ± 1.5	95	42 ± 1.5	79	43 ± 1.6	96	41 ± 1.5
Behavioral Symptoms Index	246	56 ± 1.7	92	56 ± 2.1	95	58 ± 2.2	79	55 ± 2.3	96	56 ± 2.2
Externalizing problems	246	59 ± 1.7	92	58 ± 2.1	95	60 ± 2.2	79	56 ± 2.3	96	58 ± 2.2
Internalizing problems	246	49 ± 1.4	92	50 ± 1.7	95	51 ± 1.8	79	51 ± 1.9	96	49 ± 1.8

ADHD, attention deficit–hyperactivity disorder.

a Means are adjusted for treatment group, age, caregiver’s IQ, clinic center, single parent, language, race, sex, parent’s employment, parent’s education, and concurrent blood lead level.

b LOD = 0.2 μg/L.

* *p* < 0.05, pairwise test of specified cadmium level category versus the reference (< LOD) category.

**Table 2:** Baseline and follow-up characteristics of enrolled children with detectable pretreatment cadmium.

Characteristic	Placebo (*n* = 223)	Succimer (*n* = 218)	*p*-Value
Age [years (mean ± SD)]	2.1 ± 0.4	2.1 ± 0.4	p = 0.59^a^
Weight [kg (mean ± SD)]	12.4 ± 1.8	12.7 ± 2.0	p = 0.07^a^
Height [cm (mean ± SD)]	86.4 ± 5.2	86.8 ± 5.6	p = 0.43^a^
Body surface area [m^2^ (mean ± SD)]	0.5 ± 0.1	0.5 ± 0.1	p = 0.12^a^
Clinic center [no. (%)]			p = 0.87^b^
Baltimore	54 (24)	56 (26)	
Newark	49 (22)	53 (24)	
Philadelphia	53 (24)	50 (23)	
Cincinnati	67 (30)	59 (27)	
Ethnic group or race [no. (%)]			p = 0.75^b^
White	24 (11)	27 (12)	
Non-Hispanic black	177 (79)	173 (79)	
Other	22 (10)	18 (9)	
Female sex [no. (%)]	99 (44)	93 (43)	p = 0.71^b^
English-speaking [no. (%)]	217 (97)	210 (96)	p = 0.56^b^
Parent’s education [years; no. (%)]			p = 0.62^b^
< 12	84 (38)	92 (42)	
12	103 (46)	94 (43)	
> 12	36 (16)	32 (15)	
Neither parent working [no. (%)]	132 (59)	138 (63)	p = 0.38^b^
Living with single parent [no. (%)]	162 (74)	160 (74)	p = 0.98^b^
IQ score (mean ± SD)			
MDI (analogous to 2-year IQ)	80.9 ± 12.9 (n = 217)	81.5 ± 13.8 (n = 217)	p = 0.67^a^
5-year IQ	80.7 ± 12.7 (n = 209)	79.8 ± 13.3 (n = 204)	p = 0.48^a^
7-year IQ	86.6 ± 13.4 (n = 195)	85.9 ± 12.9 (n = 178)	p = 0.62^a^
Blood cadmium [μg/L; GM (95% CI)]			
Pretreatment	0.28 (0.27–0.29)	0.29 (0.28–0.30)	p = 0.20^c^
Posttreatment	0.22 (0.21–0.24) (n = 189)	0.24 (0.22–0.26) (n = 184)	p = 0.11^c^
Blood lead [μg/dL (mean ± SD)]			
Baseline	26.1 ± 5.0	26.7 ± 5.7	p = 0.62^a^
5 year	12.0 ± 4.7 (n = 210)	13.1 ± 5.3 (n = 206)	p = 0.02^a^
7 year^d^	7.9 ± 3.7 (n = 195)	8.9 ± 4.2 (n = 172)	p = 0.02^a^

a Means were compared by *t*-test.

b Proportions were compared by Pearson chi-square test.

c Distributions were compared by Wilcoxon rank-sum test.

d One child with a blood lead concentration of 50.8 μg/dL was excluded from the analysis.

**Table 3:** GLM regression coefficients of cadmium^a^ and covariates on IQ at baseline and follow-up.

	MDI		IQ at 5 years of age		IQ at 7 years of age	
Independent variable	Coefficient	95% CI	Coefficient	95% CI	Coefficient	95% CI
Pretreatment cadmium (μg/L, ln)	3.5	−0.1 to 7.0	−1.9	−5.5 to 1.6	−2.2	−6.0 to 1.7
Treatment group			−0.5	−2.9 to 1.9	−0.1	−2.6 to 2.4
Exact age at IQ measurement (years)	−5.4	−8.4 to −2.4*	−3.2	−5.8 to −0.6*	3.6	−5.9 to 13.2
Caregiver’s IQ	0.3	0.2 to 0.4*	0.4	0.2 to 0.5^b^	0.4	0.3 to 0.5*
Center						
Baltimore	Reference					
Newark	−1.5	−5.3 to 2.2	−5.6	−9.2 to −2.1*	1.0	−3.1 to 5.1
Philadelphia	−1.7	−5.4 to 1.9	1.8	−1.7 to 5.3	6.2	2.0 to 10.5*
Cincinnati	−4.9	−8.5 to −1.4*	−4.8	−8.2 to −1.3*	−2.3	−6.4 to 1.9
Living with single parent						
No	Reference					
Yes	2.0	−1.3 to 5.3	0.7	−2.5 to 3.9	0.3	−3.0 to 3.7
Language						
English	Reference					
Spanish	1.3	−7.1 to 9.6	0.4	−7.5 to 8.2	−5.2	−12.8 to 2.4
Race						
Non-Hispanic black	Reference					
Others	1.7	−2.0 to 5.4	1.2	−2.4 to 4.7	3.0	−0.7 to 6.7
Sex						
Male	Reference					
Female	2.7	0.2 to 5.2*	−0.6	−3.0 to 1.8	−0.6	−3.0 to 1.9
Neither parent working						
Yes	Reference					
No	−0.1	−3.2 to 2.9	−0.2	−3.1 to 2.7	0.9	−2.1 to 3.9
Parent’s education (years)						
< 12	−0.1	−2.9 to 2.8	−1.0	−3.7 to 1.8	−0.5	−3.3 to 2.3
12	Reference					
> 12	−0.8	−4.5 to 3.0	−0.4	−4.0 to 3.2	2.6	−1.1 to 6.4
Concurrent lead level (μg/dL)	−0.3	−0.5 to −0.1*	−0.4	−0.6 to −0.1*	−0.3	−0.6 to −0.1*

a Includes only detectable values.

* *p* < 0.05.
